# The Formyl Peptide Receptors: Diversity of Ligands and Mechanism for Recognition

**DOI:** 10.3390/molecules22030455

**Published:** 2017-03-13

**Authors:** Hui-Qiong He, Richard D. Ye

**Affiliations:** 1 School of Pharmacy, Shanghai Jiao Tong University, Shanghai 200240, China; hhq@sjtu.edu.cn; 2 Institute of Chinese Medical Sciences, University of Macau, Macau SAR 999078, China

**Keywords:** G protein-coupled receptors, formyl peptides, inflammation, phagocytes

## Abstract

The formyl peptide receptors (FPRs) are G protein-coupled receptors that transduce chemotactic signals in phagocytes and mediate host-defense as well as inflammatory responses including cell adhesion, directed migration, granule release and superoxide production. In recent years, the cellular distribution and biological functions of FPRs have expanded to include additional roles in homeostasis of organ functions and modulation of inflammation. In a prototype, FPRs recognize peptides containing *N*-formylated methionine such as those produced in bacteria and mitochondria, thereby serving as pattern recognition receptors. The repertoire of FPR ligands, however, has expanded rapidly to include not only *N*-formyl peptides from microbes but also non-formyl peptides of microbial and host origins, synthetic small molecules and an eicosanoid. How these chemically diverse ligands are recognized by the three human FPRs (FPR1, FPR2 and FPR3) and their murine equivalents is largely unclear. In the absence of crystal structures for the FPRs, site-directed mutagenesis, computer-aided ligand docking and structural simulation have led to the identification of amino acids within FPR1 and FPR2 that interact with several formyl peptides. This review article summarizes the progress made in the understanding of FPR ligand diversity as well as ligand recognition mechanisms used by these receptors.

## 1. Introduction

The formyl peptide receptors (FPRs) are a group of G protein-coupled chemoattractant receptors that play important roles in host defense and inflammation [1,2]. They are so named because their cognate agonists are peptides bearing formylated methionine (fMet), such as those derived from bacterial and mitochondrial proteins [3,4]. The present review focuses on the FPR family members in humans and mice and their interaction with a wide variety of ligands. The reader is referred to recently published reviews on FPR signaling [5], synthetic non-peptide ligands for FPRs [6] and characterization of FPR knockout mice [7]. The reader is also referred to a general overview of FPRs and their nomenclature [2].

## 2. The Formyl Peptide Receptor Family

### 2.1. The Human Formyl Peptide Receptors

**Figure 1:**
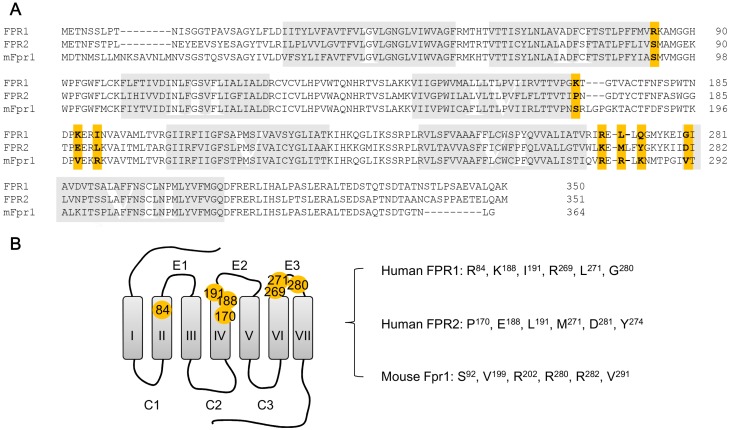
Sequence alignment and location of positive selection sites in human formyl peptide receptors (FPRs): FPR1, FPR2 and mouse mFpr1. (**A**) Alignment of the protein sequence of human FPR1, FPR2 and mouse Fpr1. Positively selected amino acid sites [14] in three FPR isoforms are shown in color; (**B**) Schematic diagram for the location of the positive selection sites in the formyl peptide receptors, based on the sequence of human FPR1. The amino acids at these positions in each of the three receptors are marked on the right.

Three genes coding for human G protein-coupled formyl peptide receptors have been cloned, including *FPR1*, *FPR2* and *FPR3* [8,9,10,11,12,13]. Among them, FPR1 and FPR2 share an overall high sequence identity (Figure 1) and certain overlapping functions.

FPR1 and FPR2 are expressed in both monocytes and neutrophils, while FPR3 is found in monocytes but not neutrophils. Besides myeloid cells, FPR1 is expressed in astrocytes, microglial cells, hepatocytes and immature dendritic cells. FPR2 shows an even wider distribution pattern than FPR1 and is expressed in a variety of non-myeloid cells including astrocytoma cells, epithelial cells, hepatocytes, microvascular endothelial cells, neuroblastoma cells, in addition to phagocytic leukocytes. In recent studies, FPR1 has been associated with anti-bacterial inflammation and metastasis of malignant glioma cells, while FPR2 is implicated in the pathogenesis of chronic inflammatory diseases such as systemic amyloidosis, Alzheimer’s disease, atherosclerosis, systemic lupus erythematosus and ovarian cancer metastasis et al. [15,16,17,18,19]. More examples and details of FPR involvement in multiple diseases are provided in recent reviews [7,20].

### 2.2. The Mouse Formyl Peptide Receptors

Since the human FPR1 gene (*FPR1*) was first cloned, orthologs have been identified in other mammalian species, including rabbits, guinea pigs, horses, rats and mice (reviewed in [2]). The genes coding for FPR members comprise a large family that implicates a complex evolutionary path with evidence of positive selection [14]. Despite overall sequence homology, these genes vary considerably in their numbers and sequences between humans and other species. For example, the mouse FPR gene family has eight known members (*mFpr1*, *mFpr2*, *mFpr-rs1*, *mFpr-rs3*, *mFpr-rs4*, *mFpr-rs6*, *mFpr-rs7* and *mFpr-rs8*) clustered on mouse chromosome 17A3.2. Among these genes, *mFpr-rs5* (*ψmFpr-rs3*) is found as a pseudogene that does not code for a functional receptor. 

Although several mouse Fpr genes are present in neutrophils, work on *mFprs* has been focused mostly on the gene products of *mFpr1* and *mFpr2*, which are widely expressed in mouse phagocytic leukocytes and show high similarity to their human counterparts. Targeted deletion of *mFpr1* or *mFpr2* renders mice more susceptible to bacterial infection without changing their viability and fertility [21,22]. Under unstimulated conditions, mice lacking *mFpr1* or *mFpr2* behave normally. However, in models of human diseases, both *mFpr1^−/−^* and *mFpr2^−/−^* mice respond differently from their wildtype littermates, indicating regulatory roles of these receptors in host defense and inflammation. Targeted gene disruption of *mFpr1* also suggested a homeostatic role for the mFpr1 in the production of glucocorticoids and anxiety-like behavior [23]. As the suggested ortholog of human FPR1, mFpr1 was identified as a low-affinity receptor for the classical formyl tri-peptide fMet-Leu-Phe (fMLF) [24], but this receptor responds well to several *N*-formyl peptides derived from other bacteria (e.g., fMIFL, fMIVIL, fMIVTLF) and mitochondria (e.g., fMMYALF) [25,26]. In general, mFpr2 is not a high-affinity receptor for either fMLF or other native formyl peptides tested so far [26]. However, it responds to endogenous peptide agonists for FPR2, including the amyloidogenic proteins SAA (serum amyloid A) [27] and Aβ_42_ [28]. Likewise, this receptor can be inhibited by FPR2-specific antagonists when expressed in neutrophil or by stably transfected cells. Besides this, mouse mFpr2 has also been reported as a receptor for F2L [29] that is a potent agonist for human FPR3 [30]. 

Published reports indicate that other members of the murine *mFpr* family are not coding for stereotypic formyl peptide receptors. In recent studies, mFpr-rs8 (*ψmFpr-rs2*) has been characterized as a constitutively expressed gene associated with mouse longevity [31]. The gene products of five other *mFprs*, including *mFpr-rs1*, *mFpr-rs3*, *mFpr-rs4*, *mFpr-rs6*, and *mFpr-rs7*, are recently described as mouse vomeronasal sensory receptors [32,33]. Phylogenetic analysis suggested that they belong to a chemosensory GPCR family, which is characterized with an olfactory function and has the ability to identify pathogenic states. Among them, the property of mFpr-rs1 is still uncertain as it is also expressed in neutrophils. It was believed that mFpr-rs1 might have some functional overlap with human FPR2 because a variant of *mFpr-rs1* was reported to encode a mouse receptor for lipoxin A4 (LXA4) [34], an eicosanoid ligand for FPR2. However, functional and pharmacology assays conducted in stably transfected cells suggested that mFpr-rs1 responds poorly to most agonists that activate human and murine FPR receptors [35]. These observations add to studies using knockout animal models and together indicate that murine Fprs (especially mFpr1 and mFpr2) share numerous structural and pharmacological properties with human FPRs, but the evolutionary correlation between them is by no means linear.

## 3. Agonists for the Formyl Peptide Receptors

The FPR family is well known for the structural diversity of their ligands, including a variety of ligands with different chemical properties and origins ranging from natural peptides to synthetic non-peptide compounds [2,5] (Table 1). FPRs are therefore classified as a group of pattern recognition receptors (PRRs) that recognize pathogen-associated molecular patterns (PAMPs) and damage-associated molecular pattern (DAMPs) [36,37,38]. 

### 3.1. Conventional Formyl Peptide Agonists for FPRs

Among all the ligands for the FPRs, *N*-formylated peptides, particularly fMLF (an *E. coli*-derived chemotactic peptide), are most often studied. fMLF is among the first characterized and also the shortest formyl peptides with full agonistic activities [39,40,41]. These peptides are initiated with *N*-formyl methionine and are generally cleavage products of bacterial and mitochondrial proteins. *N*-formylated peptides constitute the most commonly studied class of FPR agonists that trigger a variety of biological activities in myeloid cells, such as chemokinesis, chemotaxis, calcium flux, cytokine production and superoxide anion generation. Through binding with the high affinity receptor FPR1, fMLF and other *N*-formylated peptides serve as potent chemoattractants, which also include activated complements (C5a, C3a) and chemokines, in recruiting and guiding leukocytes to the site of bacterial infection and to damaged tissues. A wealth of literatures have described the proinflammatory properties of *N*-formyl peptides. As early as 30 years ago, right after the discovery of *N*-formyl peptides, studies have shown that fMLF was involved in the pathogenesis of multiple inflammatory diseases such as colitis [42,43,44], pouchitis, ulcerative colitis and Crohn’s disease [45,46] and juvenile peridotitis [47]. Studies also reported that inhalation or injection of fMLF can cause rapid neutropenia and bronchial inflammation in human and other mammals [48,49,50,51].

Human FPR2 is a low affinity receptor for fMLF and many potent formyl peptide agonists for FPR1. However, FPR2 displays relatively high affinity for *N*-formylated peptides of specific composition and longer length. For instance, FPR2 responds better to peptides carrying positive charges at the C-terminus (e.g., fMLFK, fMLFIK) than peptides with negative charges (e.g., fMLFE and fMLF) [96]. Studies also showed that FPR2 responds well to formyl peptides of microbial origin other than *E. coli*, such as fMIFL (*S. aureus*) and fMIVIL (*L. monocytogenes*) in calcium mobilization and cAMP release assays [96]. In general, most bacteria-derived formyl peptides are more potent at FPR1 than FPR2. A prominent exception is the recently described cytolytic peptides PSM (phenol-soluble modulins) that are *α*-helical peptides composed of 20–25 amino acids [97]. Although they are predominantly secreted from community-associated methicillin resistant *Staphylococcus aureus* (CA-MRSA) in formylated form, PSM*α* peptides are more selective for FPR2 than for FPR1 [98,99,100]. Another exception is mitocryptide-2, a neutrophil-activating formyl peptide produced from mitochondrial cytochromes. Mitocryptide-2 can directly bind to FPR2 and activate it with an EC_50_ of 6.9 nM in calcium flux assay, whereas it manifests no interaction with FPR1 [54]. In addition, another group of formyl peptides derived from mitochondria (fMMYALF, fMYFINILTL, and fMLKLIV) were found equally potent on FPR1 and FPR2 with EC_50_ values ranging from 10 nM to 160 nM [53]. Despite the different potency and selectivity of these formyl peptides, the *N*-formyl group is important for optimal interaction with FPR1 and FPR2. It is suggested that FPR1 is a highly effective receptor recognizing formyl peptides and mediating phagocyte functions, while FPR2 has an overall lower affinity but it can discriminate between formyl peptides with different sizes and features. In contrast, no bacterial or mitochondrial formyl peptide has been described as agonist for FPR3 by far. Noting that the prototypic FPR1 agonist fMLF is not a potent activator for murine Fprs, including mFpr1 and mFpr2, when compared to human FPR1. In agreement with the research that murine neutrophils are more susceptible to bacteria other than *E. coli* [21], mFpr1 was found more responsive to formyl peptides derived from *Listeria monocytogenes* (fMIVTLF), *Staphylococcus aureus* (fMIFL), and mitochondria (fMMYALF) [25,26]. 

### 3.2. Other Peptide Agonists for FPRs

Except for formyl peptides, a number of different peptides/proteins, including microbial and host-derived non-formyl peptides/proteins and peptides from synthetic libraries, have been identified to be agonists for FPRs. It appears that the absence of *N*-formyl group and lack of overall sequence similarity with formyl peptides mentioned above do not impair their agonistic activity at FPRs. In general, the ligand diversity is more prominent for FPR2 than FPR1.

The non-formylated peptides/proteins derived from microbe are represented by virus envelope proteins (gp41 and gp120) of the human immunodeficiency virus type 1 (HIV-1), which contains at least five FPR-recognizing peptide sequences composed of 20–37 amino acids. Most of these sequences were found more potent at FPR2 than at FPR1 and FPR3 [101,102,103,104,105], except only one is selective for FPR1 and mFpr1 [101,102,106]. A cecropin-like peptide (Hp2-20) from *Helicobacter pylori* was also identified as an agonist for FPRs [55,107]. The studies demonstrated that in *H. pylori* infection, Hp2-20 was able to induce chemotaxis of monocytes and basophils, and evoke superoxide release via signaling through FPR2 and FPR3. Recently, two antimicrobial peptides (AMPs) isolated from *Scolopendra subspinipes mutilans* were reported to elicit neutrophil chemotactic migration through FPR1 [108]. 

The host-derived non-formyl peptide agonists for FPRs are generally peptides and proteins associated with human diseases and inflammation. This group of agonists activates FPRs independently of the *N*-formyl group and they also show a preference for FPR2. A prominent example of them is serum amyloid A (SAA), an acute-phase protein that is implicated in chronic inflammation and amyloidosis. As the first identified endogenous peptide agonist for FPR [56], documented studies have shown that it exerts pro-inflammatory activities through FPR2 in phagocytes, epithelial cells and T lymphocytes, including stimulating production of inflammatory mediators and enhancing the expression of cytokine receptors [109,110,111,112,113]. Recently, questions have been raised regarding the proinflammatory cytokine-like properties of native SAA, since most studies of SAA and FPR2 are performed using a commercially available recombinant protein that contains amino acid substitutions of SAA2 in SAA1 [5,114,115]. However, research conducted with SAA1 isoforms purified from an *E. coli* expression system suggested that major SAA1 isoforms have cytokine-inducing activity and minor substitutions of amino acids affected their potency at FPR2 [116]. In vivo studies have shown Th17-induction activity of SAA in gut epithelium, although whether these activities are attributed to FPR2 or other SAA receptors remain unclear [117,118]. 

Another amyloidogenic disease-associated FPR2 agonist is the prion protein fragment PrP (106–126), which was reported to interact with FPR2 in glial cells and induce calcium mobilization, chemotaxis as well as production of pro-inflammatory cytokines [64]. In addition to SAA and PrP (106–126), other two amyloidogenic disease-associated peptides were found to be agonists for FPR2: the 42-amino acid form of Aβ amyloid peptide (Aβ_42_) and humanin. Although both peptides use FPR2 to induce migration and activation of monocytic phagocytes in the brain, Aβ_42_ and humanin display divergent roles in the development of Alzheimer’s disease: Aβ_42_ is a major cause of fibrillary formation and deposition in brain of AD patients [119,120], whereas humanin is neuroprotective on the contrary [121]. Studies suggested that humanin reduces aggregation and fibrillary formation by inhibiting Aβ_42_/FPR2 interaction in mononuclear phagocytes [121]. FPR3 and mFpr2 are also functional receptors for humanin in humans and mice, respectively [121,122].

Annexin A1, a glucocorticoid-regulated protein, and its *N*-terminal peptides (Ac2-26 and Ac9-25) are FPR agonists that have dual roles in inflammation. At high concentrations, the annexin A1 peptides fully activate FPR1 like the conventional agonists that induce pro-inflammatory responses. In contrast, at low concentrations they only show partial activity at FPR1 [123], leading to neutrophil desensitization and inhibiting neutrophil migration induced by other chemoattractants. All three FPR members are implicated in the various functions of annexin A1 peptides. Some researchers thought that these peptides use FPR2 for anti-inflammatory actions [59], while others suggested the presence of receptors other than FPR2 and FPR3 as being responsible for the resolving effects [124]. The exact mechanism for the pro-/anti-inflammatory activity of annexin A1 peptides is not entirely clear. 

The antimicrobial peptide LL-37 and its murine homolog CRAMP (Cathelicidin-Related Anti-Microbial Peptide) were identified as FPR2 agonists [61,125]. LL-37 is a cleavage product of the neutrophil granule protein cathelicidin found in leukocytes and epithelial cells. As a pro-inflammatory mediator, LL-37 activates FPR2 and evokes superoxide generation in human fibroblasts [126]. It also induces directional migration of human monocytes, neutrophils, and T lymphocytes [61]. However, LL-37 also inhibits SAA signaling in human neutrophils, including IL-8 production and chemotaxis through FPR2 [127]. LL-37 seems to be a pleiotropic peptide and its interaction with FPR2 is implicated in wound healing, cell proliferation [128], angiogenesis [129] and anti- or pro-tumorigenesis [130,131,132]. In a recent study, the proinflammatory circuits of LL-37 and leukotriene B4 was reported. In human neutrophils, LL-37/FPR2 promotes Leukotriene B4 production, which in turn elicits further LL-37 release through BLT1 (leukotriene B4 receptor 1) [133].

uPAR is the cell surface receptor for urokinase-type plasminogen activator (uPA) that is a serine protease involved in regulation of fibrinolysis, cell adhesion, migration and tissue repair. uPAR can be cleaved by different proteases, including uPA, generating soluble uPAR fragments that can be recognized by different FPR members. For instance, uPAR D2D3_88–274_, a cleaved peptide corresponding to residues from 88 to 274 of uPAR, binds to and activates FPR2 in monocytes, inducing cell migration [134]. Moreover, uPAR_88-95_ (^88^SRSRY^95^) interacts with FPR1 [62], while uPAR_84–95_ activates both FPR2 and FPR3 in basophils [63]. 

In a more recent study, a new host-derived chemotaxis agonist FAM3D, for the FPR1 and FPR2 was identified [135]. FAM3D, a cytokine-like protein, is constitutively expressed in the gastrointestinal tract with significant induction in dextran sulfate sodium (DSS)-induced colitis [135]. FAM3D was found to strongly attract the chemotaxis of human peripheral blood neutrophils and monocytes. Through functional screen of a group of chemoattractant receptors, FPR1 and FPR2 were determined to be the receptors for FAM3D. In HEK293 cells transfected to express FPR1 or FPR2, FAM3D induced significant cell chemotaxis and calcium mobilization that was desensitized by other FPR agonists (fMLF and WKYMVm), and vice versa. The activation of ERK1/2 and p38 MAPK signaling caused by FAM3D in neutrophils was blocked by an antagonist of FPR2 (WRW^4^). This study suggested that FAM3D/FPR interaction might play a role in intestinal homeostasis and gastrointestinal inflammation.

The FPR receptors also have other endogenous peptide ligands, such as cathepsin G for FPR1, chemokine CCL23 (amino acids 22–137) and its *N*-terminal fragment SHAAGtide for FPR2, a vasoactive intestinal neuropeptide VIP (vasoactive intestinal polypeptide) for FPR2, and a heme-binding protein fragment F2L for FPR3 (Table 1). In addition, exogenous allergens including house dust mite and birch pollen extracts were recently shown as being agonistic for FPR1 and FPR2. The detailed properties of these FPR agonists are discussed in previous reviews [2,5,136].

Among all the synthetic peptide agonists for FPRs, a hexapeptide, Trp-Lys-Tyr-Met-Val-D-Met-NH_2_ (WKYMVm) isolated from a random peptide library [67], was identified to be a strong activator for FPR1, FPR2 and FPR3 [68,137]. WKYMVm conjugated with FITC in the second residue Lys was shown slightly more efficacious in binding to FPR2 than to FPR1 [96]. WKYMVm is by far the most potent peptide agonist for FPR2, being able to activate FPR2 at picomolar concentrations in chemotaxis assays. WKYMVM, a derivative of WKYMVm with the substitution of l-methionine at the carboxyl terminus, becomes highly selective for FPR2 and is weakly agonistic for FPR3 [69]. Another peptide identified from library screen, MMK-1 (LESIFRSLLFRVM), is a highly selective chemotactic agonist for FPR2 [70,138]. CGEN-855A, a 21 amino acids anti-inflammatory peptide obtained from a computational platform, was identified to be agonist for FPR2 and FPR3 [139]. In another study, a tethered library was screened and a peptide with the sequence MMWLL was identified as an FPR1 agonist [140]. This peptide becomes 1000-fold more potent when the first Met is *N*-formylated, consistent with the preferential recognition of fMet-containing peptides by FPR1. In a recent study [141], a series of AApeptides, a class of peptidomimetics based on *N*-acylated-*N*-aminoethyl amino acid residues, were designed and synthesized to mimic the structure and function of the prototypic tripeptide fMLF, the reference agonist of FPR1. Three of these fMLF-mimicking AApeptides were found effective in activating FPR-expressing cells in calcium mobilization assay, albeit at concentrations above 10 µM. Certain AApeptides were shown to be more efficacious than fMLF at a higher concentration (10 µM). Similar to fMLF, these AApeptides have receptor preference for FPR1 over FPR2. 

### 3.3. Non-peptide Agonists Screened from Small Molecule Library

In comparison with peptide/protein FPR agonists, the small molecule agonists screened from chemical library are more stable and highly selective for certain FPR members. These properties make them valuable tools for detailed characterization of FPRs, which is important for a better understanding of the complex role of FPRs in vivo. FPRs are involved in a variety of signaling systems and their distribution and functions are not limited to the immune system. As a result, these small molecules screened by targeting FPRs as agonists or antagonists are expected to serve as potentially useful agents in therapeutic treatment. 

The first reported non-peptide agonist for FPR from library screening is a quinazolinone derivative named Quin-C1 [75]. Quin-C1 (4-butoxy-*N*-[2-(4-methoxy-phenyl)-4-oxo-1, 4-dihydro-2*H*-quinazolin-3-yl]-benzamide) was found highly selective for FPR2 as opposed to FPR1. It was identified as a biased agonist, as it stimulated calcium mobilization through FPR2 but did not induce substantial neutrophil superoxide generation, even at concentrations up to 100 µM. A later study revealed an anti-inflammatory role for Quin-C1 in bleomycin-induced lung injury in a mouse model [142]. The murine receptors for Quin-C1 were determined to be mFpr1 and mFpr2 [26]. Competitive binding analysis showed that Quin-C1 does not share the same recognition sites with formyl peptides in mFprs [26]. Given that Quin-C1 is non-competitive with formyl peptides, it is interesting to surmise that Quin-C1 may act at FPR2 as an ago-allosteric modulator. However, the precise mechanism for the agonistic activity of Quin-C1 requires further examination. 

Since the first synthetic non-peptide FPR agonist was reported, an increasing number of small-molecule agonists have been discovered in recent years [6]. So far, a series of structurally divergent small-molecules with high-affinity have been identified as FPR ligands via cell-based assays for high throughput screening (HTS), as well as structure-activity relationship (SAR)-directed design and synthesis [82,83,84,143,144]. In general, the identification of new molecules relies on known structures, SAR analysis and computer-aided design. Using this strategy, around one hundred FPR agonists with high potency and clear selectivity have been identified and optimized in the reported research. Based on the structures with varying scaffolds and SAR-directed evaluation, these small compounds fall into at least 10 groups: benzimidazoles, pyrazolones (e.g., Compound 43), *N*-substituted benzimidazoles, pyridazin-3(2*H*)-ones, chiral pyridazines, *N*-phenylureas, chiral 3-(1*H*-indol-3-yl)-2-[3-(4-nitrophenyl)ureido] propanamides, 2-(*N*-Piperazinyl)acetamide derivatives, quinazolinones (exemplified by Quin-C1), and other derivatives (reviewed by [6]). Among these molecules, the most potent FPR1-selective agonist was identified to be a polyphenylure derivative with an EC_50_ of 131 nM in calcium flux assays and a Ki of 4 nM in binding assays. This compound, [3-phenyl-1-((*R*)-1-(3-phenyl-1-((*S*)-1-(3-phenylureido)propan-2-yl)ureido)hexan-2-yl)-1-(4-phenylbutyl)urea], coded as 1753-103 is also the most potent synthetic non-peptide agonist for FPR1 known to date [84]. In addition to 1753-103, other chemical agonists with high FPR1 specificity, such as the benzimidazole derivates [145] and the pyridazinone-based compounds [146], have been described, although they act with EC_50_ above micromolar (>1.5 μM). Compared with FPR1, FPR2 have more specific and potent agonists identified from screening of compound library and through rational design. Currently, more than 10 FPR2-slective agonists have been reported to have an EC_50_ in the nanomolar range in functional assays, including three pyrazolone derivatives [78], an *N*-substituted benzimidazole derivative [77], an *N*-phenylurea derivative [147], a chiral *N*-phenylurea derivative [148], two benzodioxole derivatives of *N*-phenylureas [148], six chiral ureidopropanamido derivatives [82,83], a phenylsubstituted urea compound [143], a compound with a unique 2-(4-phenyl-5-((phenylamino)methyl)-1,2,4-triazol-3-ylthio) acetamide scaffold [143], and a pyrrolidine bis-diketopiperazine derivative [84]. As expected, there are numerous agonists with dual specificity (FPR1/FPR2) (reviewed by [6]). Noting that a pyrazolone derivative, designated in most publications as “Compound 43”, was first identified as a FPR2-specific agonist [78], but it was redefined to be a mixed FPR1/FPR2 agonist in an independent screen recently [79]. It is not clear for most of the reported small molecule agonists whether they interact with murine formyl peptide receptors since they are designed and screened to target human FPRs and murine FPRs have distinct properties compared to their human counterparts. 

### 3.4. Host-derived Lipid and Lipopeptide Agonists

Lipoxin A4 (LXA4), derived from arachidonic acid, has highly potent anti-inflammatory and pro-resolving activities based on in vivo studies [149,150,151,152]. This eicosanoid was reported to directly bind to FPR2 and a variant of mouse mFpr-rs1 [34,74,149,153]. LXA4 was shown to have a high affinity (*K*_d_ of ~ 1.7 nM) in binding to FPR2-expressing Chinese hamster ovary cells [74]. Despite the high-affinity binding, LXA4 is an atypical ligand for FPR2 as it cannot activate proinflammatory activities such as chemotaxis, enzyme release and ROS production. LXA4 also fails to activate FPR2-dependent signaling, such as calcium mobilization, in transfected cell lines [154,155,156]. These findings do not support the original report that LXA4 could stimulate GTPase activity in FPR2-expressing CHO cells [74]. It appears difficult to determine the role of LXA4 as an FPR2 agonist without establishing a standard in compound preparation. Moreover, FPR2 specific antagonists, stereoselective analogs, antibodies, and transgenic approaches are required in further studies, and the interaction of LXA4 with other receptors such as the cannabinoid receptor CB1 [157], aryl hydrocarbon receptor (AhR) [158] and estrogen receptor [159] will have to be considered. 

Resolvins, protectins and maresins are novel pro-resolving mediators that are biosynthesized from omega-3 fatty acids, including docosahexaenoic acid (DHA) and eicosapentaenoic acid (EPA). These lipid mediators block neutrophil recruitment, promote monocyte activation, and enhance macrophage phagocytosis. Resolvin D1, a biosynthetic product from DHA, was reported to activate FPR2 and GPR32, an orphan GPCR. Similar with LXA4, Resolvin D1 cannot evoke calcium mobilization through FPR2 or GPR32. The interaction of Resolvin D1 with these GPCRs was examined by a GPCR-β-arrestin-coupled system [160]. Selective knock down or inhibition of FPR2 reduces the Resolvin D1 response, such as macrophage phagocytosis [160] and salivary epithelium migration [161]. Resolvin D1 and FPR2 are also implicated in pulmonary inflammation [162] and obesity [163]. 

Oxidized low-density lipoprotein (oxLDL), an atherogenesis associated molecule, was recently found to stimulate macrophages via FPR2 signaling [164]. The oxLDL-induced foam cell formation and TNF-α production could be inhibited by an FPR2 antagonist WRW^4^, but not affected by an FPR1 antagonist. In FPR2-expressing RBL-2H3 cells, oxLDL also stimulated significant calcium mobilization and chemotaxis [164]. In another study, the mimetic peptide L-37pA (Table 1) of apoA-I, a major component of circulating high-density lipoprotein (HDL), was reported to induce calcium flux and chemotaxis through FPR2. L-37pA is probably a biased agonist of FPR2 as it fails to induce superoxide generation in human neutrophils at a concentration up to 20 μM [71]. D-37pA, the D-stereoisomer of L-37pA, blocks L-37pA signaling and induces chemotaxis but not calcium flux. It is unclear whether D-37pA is an anti-inflammatory antagonist of FPR2. It has been noted that apoA-I itself and apoE, another important component of HDL, are described as being anti-inflammatory, as they suppress chemotaxis of leukocytes in the airway.

Currently, there has been an increasing interest in a group of newly emerging molecules that modulate GPCR activity by entirely novel mechanisms. These molecules, known as pepducins, comprise a lipid moiety conjugated with a peptide that is derived from a sequence of the cytoplasmic loops or the C-terminal tail of the target GPCR [165,166]. With the facilitation of the lipid moiety, pepducins are thought to pass across plasma membrane and anchor in the cytosolic interface to activate or inhibit signaling of receptors [166]. These lipidated peptides have been proposed as allosteric modulators, because the binding sites and action modes are different from those of conventional ligands that generally interact with extracellular domains of receptors [5,166]. Despite that the ‘allosteric modulation’ mechanism is not yet fully clarified, several receptor-specific pepducins have been identified, including an FPR2 agonistic pepducin F2Pal16. F2Pal16 is composed of palmitic acid linked to a 16-mer peptide with sequence identical to the third intracellular loop of FPR2. F2Pal16 was found to activate FPR2, like conventional FPR2 agonists, in phagocytes and stably transfected HL-60 cells [72,73]. A shorter variant F2Pal10, with higher potency compared to F2Pal16, was shown to act as a partial agonist for direct activation of FPR2 but as a full agonist for cross-talk triggered hPFR2 reactivation generated by PAF receptor and ATP receptor (P2Y2R) [73,167]. It is noteworthy that pepducins are not always specific for their designated target receptor. For example, the pepducins P2Y2PalIC2 and P2Y2PalIC3 that contain sequences of the second and third intracellular loops of P2Y2R, were identified to be FPR2 agonists and could activate neutrophils with responses inhibited by FPR2 antagonists but not P2Y2R antagonist [168]. In a recent study, a pepducin (ATI-2341) designed for CXCR4, a chemokine receptor, was found to activate neutrophil functions through FPR2-dependent signaling [169]. The selectivity of pepducins for the FPR2 appears not entirely relying on the segment of the cognate receptor. Specific sequence and amino acid are required for the action of FPR2 pepducins. It is also notable that pepducin derived from another intracellular loop has no effect [168] and pepducin with an amino acid substitution lost all activity [73]. More efforts are needed to delineate the mechanisms of action by which pepducins recognize and modulate FPR2. Of interest, although FPR1 and FPR2 share very similar sequence in the intracellular loops, there are no FPR1 specific pepducins so far. This suggests not only the divergence in the G protein signaling triggered from cytosolic domains of FPR1 and FPR2, but also the presence of different forms (such as homo- or heterodimers) involved in the activation of these two major human FPRs. However, this speculation needs verification. It also remains a question whether the pepducins mentioned above can interact with murine Fprs.

## 4. Antagonists for the Formyl Peptide Receptors

As FPRs are potentially attractive drug target, molecules that inhibit cell responses induced by FPR agonists are thought to have therapeutic value for FPR-related diseases. These molecules, generally originating from natural peptides and secondary metabolites, can interact with FPRs directly or interfere with their downstream signaling pathways. In addition to drug development, radio-labeled FPR antagonists can be used as tracking probes for in vivo imaging of infiltrating neutrophils [170,171].

### 4.1. Natural Peptides and Analogs

The first FPR antagonists were obtained by modifying the amino termini of the classical tripeptide fMLF. Early studies have shown that the *N*-formyl group is important for the formyl peptides to be recognized by FPR1. Freer et al. substituted this group with tert-butyloxycarbonyl group (*t*-Boc) and found the resulting peptide (*t*-Boc-MLF, Boc-1) exhibiting FPR1 antagonistic properties [40]. The switch from an agonist to an antagonist was also observed when other carbamates (such as iso-butyloxycarbonyl and benzyloxycarbonyl group) were used to replace the formyl group at the amino terminus [172]. Further C-terminal modification of iso-butyloxycarbonyl-MLF (*i*-Boc-MLF) did not alter the antagonistic potency, implying the different roles of amino and carboxyl terminus in conferring the activities to these peptides. The *t*-butyloxycarbonyl analog of formylated peptide f-FLFLF (*t*-Boc-FLFLF, Boc-2) is another antagonistic peptide that potently inhibits neutrophil NADPH-oxidase activity induced by fMLF [40,173,174]. There is evidence that Boc-1 and Boc-2 begin to lose their receptor preference to FPR1 at high concentrations (>5 µM) and display inhibitory effects on FPR2 and even the complement component 5a (C5a)-induced responses [174]. Thus, despite their fairly specific activity at low concentrations, these Boc peptides may be classified as pan-antagonists for FPRs. However, other disagree because receptor overlap was seen at high concentrations of all antagonists [5]. In a recent study, optimization at the Phe residues of Boc-2 following Ala-scanning led to a more potent FPR1-selective antagonist [175]. Nevertheless, among the available formyl peptide-derived antagonists, Boc-1 and Boc-2 are still most potent and commonly used in experiments. 

Chemotaxis inhibitory protein of *S. aureus* (CHIPS) is another native protein (14.1 kDa) with FPR antagonistic activity. The full length CHIPS (121 amino acids) was first reported to be an antagonist for both FPR1 and the C5a receptor [176]. However, the *N*-terminal peptides FTFEPFPTNEEIESN and FTFEPF (the first six residues) display only anti-FPR1 properties [85], suggesting a mechanism of *S. aureus* invasion through inhibiting fMLF-induced neutrophil chemotaxis. Accordingly, neutralizing antibodies against CHIPS may be useful for treating *S. aureus* infection [177] given the fact that CHIPS can bind to FPR1 with high affinity (*K*_d_ ~ 35 nM) [176]. In addition, another *S. aureus*-derived 105-amino acid protein FLIPr (FPR-like 1 inhibitory protein), was reported to selectively inhibit the binding of and activation by FPR2 agonists [178].

Several peptides derived from viruses were also found inhibitory to FPR activation. These peptides include a retroviral p15E-derived hexapeptide (LDLLFL) that can inhibit fMLF-induced response in monocytes and granulocytes [179], and a coronavirus 229E-derived 12-mer peptide (ETYIKPWWVWL) that was identified as a potent antagonist of FPR1 with a K_i_ of 230 nM [180]. Additional peptides isolated from HIV-1, HIV-2, severe acute respiratory syndrome coronavirus and Ebola virus were reported as FPR1 antagonists with lower affinities [180]. It is interesting that *N*-formylation of ETYIKPWWVWL transforms this 12-mer peptide into an FPR1 agonist, which is consistent with the notion that modification of size and shape at the amino terminus can change the FPR agonistic/antagonistic property of some peptides. 

Among the antagonistic cyclic peptides from natural sources, cyclosporin H (CsH) is one of the most potent and specific FPR1 antagonists. CsH and another cyclic undecapeptide CsA are derived from peptide metabolites of the fungus *Tolypocladium inflatum* [87,144,174]. Several cyclosporins were synthesized based on CsH and SAR analysis [181]. Competition assays conducted with radionuclide binding or *N*-acetyl-β-d-glucosaminidase release assay demonstrated that cyclosporin H and its derivatives are potent FPR1 antagonists with nanomolar IC_50_ values [181,182]. These compounds were shown to be specific to FPR1 only at low concentrations. The blocking effect on FPR2 signaling is also observed with CsH at high concentrations (>2.5 μM) [174]. It was reported that CsH could attenuate mouse acute inflammation evoked by cigarette smoking [183]. Another study revealed that administration of CsH reduced opioid peptide secretion and analgesia associated with neutrophil activation during *Mycobacteria butyricum* infection [184]. CsH and derivatives are anticipated to be useful in the treatment of airway, gastrointestinal tract and skin inflammatory disorders associated with neutrophil and eosinophil infiltration. 

An endogenous allergen uteroglobin (UG) was previously identified as an FPR2 antagonist. UG, known as Clara cell 10 kDa protein (CC10) in humans, is an anti-inflammatory and anti-chemotactic protein that is constitutively expressed in mammalian airway epithelium. Before molecular cloning of FPR, UG was found to inhibit fMLF-induced chemotaxis of monocytes and neutrophils [185]. After molecular cloning of the FPR genes, the high affinity binding of UG to FPR2 was determined with a *K*_d_ of 33 nM in HEK293 cells stably expressing FPR2 [86]. The interaction of UG with FPR2 inhibits SAA expression and SAA-driven inflammation [86,186]. The activity of UG at FPR1 or other receptors has not been examined yet, thus the selectivity of this allergen is not fully understood. 

An additional natural peptide with antagonistic activity is a urokinase receptor-derived cyclic peptide. Note that the linear form of this peptide ^88^Ser-Arg-Ser-Arg-Tyr^92^ is actually a dual agonist for FPR1 and FPR2 [62]. The cyclized SRSRY was reported to inhibit the binding of fluorescence labeled fMLF to FPR1-expressing RBL-2H3 cells and it inhibits fMLF-directed monocyte migration with a picomolar IC_50_ [88]. However, the activity of cyclized SRSRY on FPR2 was not verified in the study. An extract of secondary metabolites from a marine *Bacillus* sp. was also found to have FPR antagonistic properties [187]. The precise molecule responsible for the inhibitory activity has not yet been identified. Other endogenous non-cyclized peptides with antagonistic activity include spinorphin (LVVYPWT) [188] and sarpopeptate, an aurantiamide isolated from plants and fungi [189]. Spinorphin is an FPR1 antagonist [188,190]. Sarpopeptate and its dipeptide derivatives were shown to have potent inhibitory effects on fMLF-stimulated neutrophil responses [191,192] and exhibit a receptor preference for FPR1 over FPR2 [193]. However, the receptor specificity needs further identification because of the lack of data for binding analysis. 

### 4.2. Other Peptide Antagonists

In a ligand screen of hexapeptide libraries, several peptides were found to inhibit the binding of FPR2 agonist WKYMVm. The peptide WRWWWW (WRW^4^) is the most potent of these peptides with antagonistic activity against FPR2 agonists [89]. In addition, WRW4 is the first FPR3 antagonist that can completely inhibit the activation of FPR3 by F2L in FPR3-transfected HEK293 cells [194]. It inhibits F2L-induced response in mature monocyte-derived dendritic cells, which express FPR3 but not other FPRs.

Recently, a cell permeable peptide of 10 amino acids conjugated with a rhodamine group (RhoB-QRLFQVKGRR, PBP10) was shown to have potent inhibitory activity on FPR2. The peptide sequence of PBP10 is derived from PIP2-binding domains of the cytoskeletal protein gelsolin, and the rhodamine group is essential for the peptide to pass through plasma membrane and exert antagonistic activity. This rhodamine-linked decapeptide can completely inhibit ROS production mediated by FPR2 but not by FPR1 [90]. In a shorter form, RhoB-QRLFQVG maintains potent antagonistic activity for FPR2 and displays the ability to partially inhibit FPR1. Since PBP peptides bind to the intracellular domain of receptors, the difference in length may reflect divergent structural features of the cytosolic side of FPR1 and FPR2. It is notable that PBP10 is not an FPR2-specific antagonist as it also affects non-FPR2 mediated signaling [90]. 

### 4.3. Lipopeptide Antagonists 

The pepducin F1Pal16, derived from a segment of the third intracellular loop of FPR1, was found to inhibit FPR2-mediated cellular response, but had no effect on FPR1 signaling [91]. The FPR2 antagonistic property of F1Pal16 was confirmed by selective inhibition in the binding of a conventional FPR2 agonist (Cy5-WKYMVM). This is not the first pepducin designed to target another GPCR but instead was found to hijack FPR2; however it is the first FPR pepducin with antagonistic activity. 

Pam-(Lys-βNSpe)6-NH2, a lapidated α-peptide/β-peptoid oligomers was recently found to be a cross-species antagonist with selectivity for FPR2 and mFpr2 [92,93]. Pam-(Lys-βNSpe)6-NH_2_ belongs to a group of immunomodulatory host defense peptides (HDPs), however, unlike most HDPs, this synthetic HDP mimic is resistant to proteolysis. It was revealed that both the lipid and peptidomimetic portions are important for its immunomodulatory function. Pam-(Lys-βNSpe)6-NH_2_ can directly bind to FPR2 and prevent the binding of fluorescently labeled FPR2-specific agonist (Cy5-WKYMWM) but not the FPR1-specific agonist (FITC-fNLFNYK) [92]. The inhibitory potency of Pam-(Lys-βNSpe)6-NH_2_ is close to that of PBP10. Both FPR2 antagonists are membrane permeable and display a reversible inhibition pattern [92]. Despite the similarity, their action mechanisms appear divergent, because the rhodamine group in PBP10 cannot be exchanged for hexadecanoic acid in the HDP mimic peptide [90]. It was suggested that Pam-(Lys-βNSpe)6-NH_2_ and its structural analogs are allosteric modulators of FPR2, because they can inhibit neutrophil responses induced by the FPR2 pepducin F2Pal10, which is assumed to act in a different way from conventional FPR2 agonists by anchoring to the intracellular loop of the receptor [73]. However, like other FPR2 antagonists such as WRW^4^, PBP10 and FLIPr were also found to inhibit F2Pal10 at FPR2, an assumption requiring further verification. 

### 4.4. Non-Peptide Molecules and Derivatives

As FPRs are potential drug targets, the search for novel FPR antagonists/inhibitors with high affinity and selectivity remains one of the fundamental aims of biomedical research in this field. The first reported FPR1 competitive antagonist is a non-steroidal anti-inflammatory drug (NSAID), Sulfinpyrazone and its derivative 1,2-diphenyl-4-(3-(1-naphthyl)-propyl)-3,5-pyrazolidinedione (DNP) [195,196]. However, Sulfinpyrazone is a low affinity (*K*_i_ = 14 μM) and non-specific ligand at FPR1 [197,198]. In recent years, numerous small molecule compounds have been discovered to have antagonistic/inhibitory effects using HTS of either mixture-based combinatorial libraries or natural product-based drug designs combined with SAR analysis and computational modeling. Although there are hundreds of antagonistic/inhibitory small molecules in published reports, the fully characterized chemical antagonists with high potency (*K*_i_ < 10 µM) are no more than 10 compounds after excluding those without binding analysis and/or agonistic activity assays. 

The 4*H*-chromone compound (6-hexyl-2-methyl-3-(1-methyl-1*H*-benzimidazol-2-yl)-4-oxo-4*H*-chromen-7-yl acetate) was identified as the most potent FPR1 antagonist (*K*_i_ ~ 100 nM) among the related synthetic and natural isoflavones. This isoflavone and analogs were found to suppress agonist-stimulated calcium flux and chemotaxis of neutrophils with IC_50_ values of 0.02~20 μM [94]. The competitive analysis showed that they are specific for FPR1 and did not inhibit FPR2-, FPR3-, CXCR1- or murine mFpr1-dependent responses. They have been also shown to be inactive as direct agonists of FPR1/FPR2 and mFpr1 [94]. A hederagenin SMG-1 [3-*O*-(3,4-Odi-acetyl-α-l-arabinopyranoside)-(1 → 3)-α-l-rhamnopyranosyl-(1 → 2)-α-l-arabinopyranoside], a saponin isolated from *Sapindus mukorossi*, was reported to block binding of fluorescently labeled FPR1 ligand fNLFNYK (*K*_i_ < 10 µM) and inhibit FPR1-mediated response in neutrophils and FPR1-expressing HL-60 cells [199,200]. It has been verified that SMG-1 has no direct agonist effects. In another study, a lignin PP-6 [(2*R*,3*R*)-2-(3′,4′-dihydroxybenzyl)-3-(3′′,4″-dimethoxybenzyl) butyrolactone], isolated from *Piper philippinum*, was found to inhibit fMLF-induced neutrophil response and block FITC-fMLF binding to neutrophil with a *K*_i_ of ~1.5 μM. In the studies of a pharmacophore model of FPR1 antagonists and molecular docking, PP-6 displays impressive properties of FPR1 antagonism [200]. However, it is difficult to consider PP-6 a competitive antagonist for FPR1 based on the experimental data because the antagonistic effect of PP-6 appears non-competitive and reversible [201]. Further studies are also necessary to address the specificity of this lignin. 

By screening combinatorial compound libraries, a pyrolidine bis-diketopiperazine-based compound 1754-31 was recently identified as the most potent non-peptide FPR2 antagonist with a *K*_i_ of 1 nM and an IC_50_ of 81 nM in calcium mobilization assays. The compound 1754-31 [(*R*)-4-(cyclohexylmethyl)-5-(4-hydroxybenzyl)-1-((*R*)-1-((*S*)-2-(((*S*)-6-isopropyl-2,3-dioxopiperazin-1-yl)methyl)pyrrolidin-1-yl)-3-(naphthalen-2-yl)propan-2-yl)piperazine-2,3-dione]) showed no agonistic activity at concentrations up to 12 μM [84]. A quinazolinone derivative was previously reported to suppress FPR agonist-stimulated inflammatory responses in vitro and in an in vivo model [96]. This Quin-C1 related quinazolinone, namely Quin-C7, was proven to have no agonist activity. It could displace [^125^I]WKYMVm binding to FPR2 with an estimated *K*_i_ of 6.7 μM. It did not inhibit the binding of [^3^H]fMLF to FPR1 at concentrations up to 100 µM, indicating a high preference for FPR2 over FPR1 [95]. Quin-C7 was described as an FPR2-specific antagonist, albeit its activity at other inflammation-related receptors (such as C5aR and CXCR) has not been fully examined. Additional FPR2-specific antagonists with an 2-phenylimidazo[1,2-*a*]pyrimidine scaffold were discovered through the use of a high-throughput HyperCyt flow cytometric platform [202,203,204,205]. They were reported to competitively bind to FPR2 with *K*_i_ values of 0.21, 1.8 and 3.2 μM. The subsequent functional analysis revealed no direct agonistic effects in one of the most potent compounds [200]. More information and analysis are provided in recent reviews [6,200]. 

In summary, future studies of binding properties and off-target effects are needed to determine the bona fide antagonists among compounds that have been identified to suppress FPR agonist-stimulated cell responses in functional activity assays. Moreover, most of the antagonists mentioned above have been characterized as human ligands, and their potency and specificity for the murine FPRs remain to be determined.

## 5. Structural Basis for Ligand Detection

Despite the lack of crystal structures, efforts in understanding FPR structure–function relationship has been propelled with approaches of molecular modification (including construction of receptor chimera and site-directed mutagenesis) and computational docking studies. Several clusters of key residues have been identified to be crucial for the interaction between FPRs and various modulators with highly divergent profiles and properties. Mutational studies have confirmed the individual functions of some of these residues in FPR–ligand interaction [96].

### 5.1. Binding Sites for Formyl Peptides

**Figure 2:**
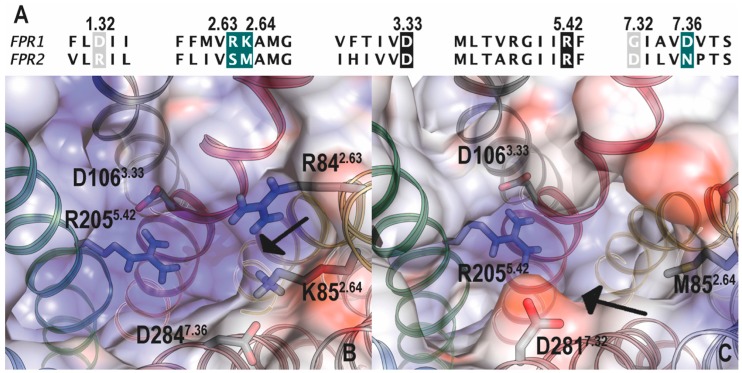
Comparison of FPR1 and FPR2 in selected regions. (**A**) Sequence alignment of FPR1 and FPR2. Residues referenced in the manuscript are boxed. Conserved side-chains between both receptors are shown in black, and major differences in amino acids are highlighted in green for *N*-formyl peptide binding to FPR1) or gray (for *N*-formyl peptide binding to FPR2). B and C, molecular electrostatic potential on the inner surface of the binding cavity of a computational model of FPR1 (**B**) and FPR2 (**C**), viewed from top of the receptors. FPR1 and FPR2 were modeled using the structure of the CXCR4 chemokine receptor as template. Arrows show key positive area in FPR1 due to Arg^84^ and Lys^85^, and negative area in FPR2 due to Asp^281^ sequence alignment and location of positive selection sites in FPR1, FPR2 and mFpr1 (see also Figure 1). Reproduced from [96] with permission.

Using receptor chimeras constructed by domain swapping between FPR1/FPR2 [206] and FPR1/C5aR [207], early studies demonstrated that the first, second and third extracellular loops and adjacent membrane regions seem to be important for recognition of fMLF. This approach was also taken to study chemotaxis mediated by FPRs [208]. Following these studies, point mutations were introduced into both FPRs, resulting in the identification of multiple charged amino acids that are important for the interaction with fMLF [209,210,211,212]. These non-contiguous residues include Arg^84^, Lys^85^, Arg^163^, Arg^205^, and Asp^284^ in FPR1 (Figure 1). Among them, Lys^85^ in the second transmembrane segment (TM2) and Asp^284^ in TM7 were proven essential for the high-affinity binding of fMLF [212,213]. It is suggested that binding of fMLF to FPR1 disrupts the electrostatic interaction between Lys^85^ and Asp^284^, which might contribute to the receptor activation [212,214,215] (Figure 2). The lack of a ‘salt bridge’ formed by Lys/Asp in FPR2 (becoming Met and Asn in corresponding positions) is responsible for the low affinity binding of fMLF and most formyl peptide, although it cannot explain the relatively effective recognition by formyl peptides of other compositions and lengths. 

**Figure 3:**
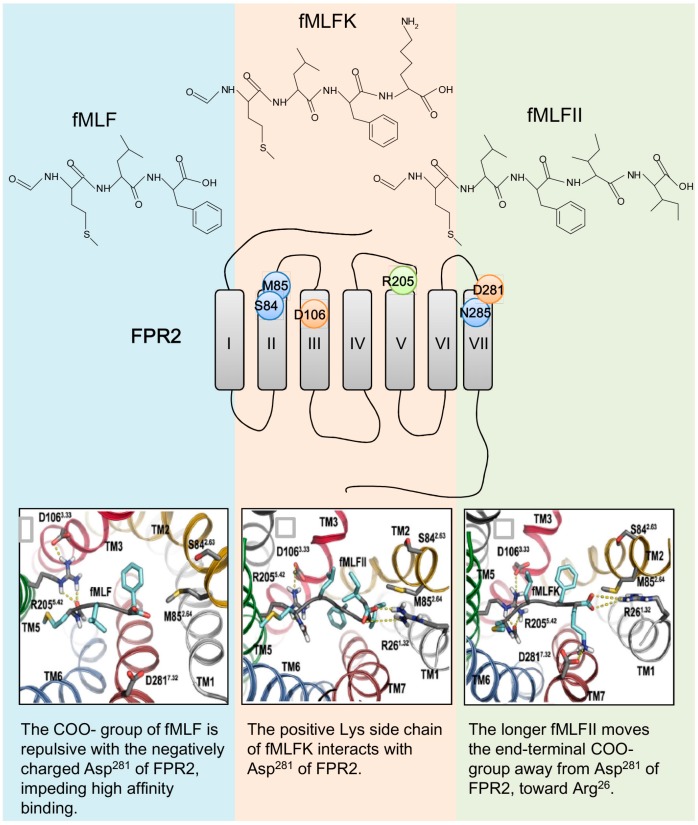
CXCR4-based homology models of FPR2 (middle) in complex with three formyl peptides of different composition (top). The lower panel show the molecular interaction of FPR2 residues with the side chains of fMLF (left), fMLFK (middle) and fMLFII (right) in a computational model of FPR2. The lower panels were adapted from [96] with permission.

Using a similar approach of site-directed mutagenesis aided by computer modeling, another charged residue Asp^281^ in FPR2 (Gly^280^ in FPR1) was found to be crucial for the interaction of FPR2 with certain formyl peptides that contain a positively charged C-terminal residue (Figure 3), such as Lys in fMLFK [96]. In this case, the formation of ‘salt bridge’ seems still important because the D281G substitution of FPR2 alone failed to restore the potency of fMLF to the level in FPR1 [96]. Studies have also shown that longer formyl peptides with α-helical and amphipathic properties favor FPR2 over FPR1 [52,216,217]. A simple explanation is that the binding pocket of FPR2 is larger and deeper than the relatively shallow and narrow one in FPR1, thus providing an added advantage in accommodating longer peptides. 

### 5.2. Binding Sites for Other FPR Ligands

To understand the structural basis for the selective activation of FPR2 by pro-resolving modulators, chimeric receptors were constructed between FPR2 and other receptors, including FPR1 and BLT1. The study using these chimeras demonstrated that TM7 and adjacent regions of FPR2 are essential for the recognition of LXA4. In contrast, the extracellular loops were required for high affinity binding for pro-inflammatory peptide agonists such as MMK1 and the MHC peptides. In addition, conserved *N*-glycosylation sites in FPR2 (Asn^4^ and Asn^179^) were also implicated in the interaction with these peptides but not with LXA4 [2,218]. In another study, a computational model of FPR1–annexin peptide interaction was built and it suggested that the transmembrane portions (TM2, TM5, TM6, TM7) of the binding pocket of FPR1 is important to optimal binding of the *N*-terminal residue in Ac-^9^QAWF^12^, the core structure of active annexin peptides [219]. Using the same set of chimeric FPR1/FPR2 receptor [206] stably expressed in HEK293 cells [208], the *N*-terminal region and the second extracellular loop of FPR2 were identified as required for annexin A1-mediated signaling [220].

Chimeric receptors were also constructed to dissect the mechanisms of action by which cell-penetrating lipopeptides (e.g., pepducins and PBP10) use unconventional access to modulate FPR2. It was suggested that the pepducins F2Pal16 and F2Pal10 act primarily through the third intracellular loop of FPR2. The third intracellular loops of FPR1 and FPR2 differ in only two amino acids. However, a substitution of these amino acids from FPR1 to FPR2 did not affect the selectivity of these pepducins [73]. Another study focused on PBP10, an antagonist against FPR2 agonists, suggested that neither the third intracellular loop nor the presumed signaling cytoplasmic tail of FPR2 alone is responsible for the specific inhibition of PBP10 [90]. Clearly, the current knowledge on the action mode and binding sites of these lipopeptides is still very limited. Perhaps new strategies should be exploited to understand the structure–function relationship of FPR in association with these allosteric modulators before crystal structures become available.

### 5.3. Docking Studies 

Due to the lack of crystal structures for FPRs, homology models and molecular docking analysis based on the mutagenesis/chimera studies represent an alternative approach for explaining ligand binding and receptor activation. The current homology models of FPR receptors (including FPR1 and FPR2) are generally created by using the crystal structures of two classes of receptors: (1) the bovine rhodopsin receptor, that has a relatively low sequence identity to FPRs (~20%) and was selected as a template because of its higher resolution (2.2 Å) [221]; (2) the CXCR4-based templates, usually CXCR4 alone, which has a higher sequence identity with FPRs (28%) but has a lower resolution (3.2 Å) compared to that of rhodopsin [222]. A dual template strategy (CXCR4 and μOR opioid receptor) was developed in a recent docking study focused on FPR2 and non-peptide ligands [223]. 

Despite questions about the low similarity of rhodopsin receptor with FPRs, several interesting points have been made by using the homology models based on this receptor. The homology model derived from the rhodopsin template shows that the FPR1 ligand binding site comprises two channels, two cavities, and the bottom [145]. Several amino acids are found to be involved in the interaction of FPR1 with agonists, including Asn^192^, Thr^199^, Arg^205^, Tyr^257^, and Thr^265^ [80,145,146,212]. Docking studies with FPR1 non-peptide antagonists (including the bile acids DCA and CDCA) suggested that Thr^177^, Tyr^257^, and Thr^265^ are involved in the formation of three stable H-bond interactions with DCA and CDCA in the docked pose [224,225]. A pharmacophore model based on best docking poses of four FPR1 antagonists including CsH described three anchor points: two acceptors for H-bonding and one hydrophobic point [172,224,226]. In comparison with the agonists, FPR1 antagonists are characterized as containing more OH groups, which can serve as H-bond donors and/or acceptors upon binding to FPR1 [6]. However, the three-point model was further proved to be not only fitted by FPR1 antagonists but also by FPR1 agonists [147,227]. Thus, this model may be helpful in prediction for structures of FPR1 ligands without discriminating agonists and antagonists. In another study, a CXCR4-based homology model of FPR1 suggested an important role of water molecule in discrimination between FPR1 agonists and antagonists [228]. In a comparative model, the potent antagonist Boc-1 was found to interact with FPR1 by forming H-bonds with Arg^84^ and Lys^85^, similarly to the interaction between fMLF and FPR1. However, Boc-1 directly forms an H-bond with Asp^284^ while water molecule mediates H-bonding between the fMLF carbonyl group and Asp^284^ [228]. 

The rhodopsin-based model with five FPR2 agonists described the binding pocket of FPR2 as dumb-bell shaped, with a large cavity and a small cavity linked by a narrow channel. The proposed model comprises three sub-pockets, the pharmacophore sub-pocket I (within the small cavity) surrounded by positive residues, and the hydrophobic sub-pockets II and III (within the large cavity) surrounded by polar residues [82]. The FPR2-specific peptide agonist WKYMVM fits well with this model by occupying all three sub-pockets, with the *N*-terminal indole moiety located in sub-pocket I in the best docked pose [6]. In another docking simulation, residues His^102^, Val^105^, Asp^106^, Leu^109^, and Trp^254^ were identified as being critical for agonist binding [229]. In a more recent study, comparative docking analysis was performed on a homology model of FPR2 that used CXCR4 and μOR as a dual template [223]. The model proposed that three hydrophobic clusters are important in binding non-peptide ligands, including agonists and antagonists. The first cluster is formed by TM2 and TM3 while His^102^ is crucial in the interaction with docked ligands. The second cluster is located between TM6 and TM7 with Phe^257^ as the key residue in binding to aromatic groups of docked compounds. The third cluster is buried in the protein consisting of residues Phe^74^, Val^105^, Phe^257^ and Phe^292^ [223]. The docking model suggested that the proper orientation is required for penetration into the binding site: the peptide ligands are placed upright while non-peptide ligands prefer a more horizontal position. These findings have yet to be substantiated by mutational studies. Still, the differences in binding modes between antagonists and agonists to FPR2 are not fully understood. To date, there are no reports describing modeling of FPR3 and mouse mFprs. 

## 6. Conclusions

The FPRs have evolved to be a class of receptors that not only recognize bacteria-derived formyl peptides but also ligands with drastically different structures, including non-formyl peptides of microbial origins, endogenous peptides and synthetic small molecules. Because of these features, the FPRs are highly ‘promiscuous’ in terms of ligand recognition. How FPRs recognize these different ligands remains unclear at present without available crystal structures. Molecular docking and computer simulation approaches combined with site-directed mutagenesis, however, have provided clues to our understanding of how formyl peptides interact with FPR1 and to a lesser extent, FPR2. Over a very long period of time, adaptive evolution has occurred in FPRs, especially FPR1, with positive selection for receptor interaction with the bona fide or orthosteric ligands. Indeed, several residues involved in H-bonding with amino acids in formyl peptides are located within the predicted binding sites undergoing positive selection, suggesting that human FPR1 is under selection pressure for its binding of formyl peptides. As for other FPR1 agonists as well as for FPR3, the picture remains unclear. There is no doubt that some agonists for the FPRs have been in existence only recently and they are likely candidates for allosteric or ago-allosteric modulators of the FPRs. With respect to FPR2, it apparently has diverged from the evolutionary path taken by FPR1, resulting in a different recognition profile including binding of longer formyl peptides and a wide range of endogenous and microbial peptides. A better understanding of the mechanisms by which FPRs interact with their ligands will help to sort out agonists and antagonists that are crucial for the biological functions of these receptors, and to speed up identification of therapeutically important molecules.

## Figures and Tables

**Table 1:** Structures and sequences of representative FPR ligands. The table shows selected FPR ligands in different categories, based on their origins and chemical structures. Ac = acetyl; Me = methyl; Ph = phenyl; Pam = palmitoyl; N.D, binding affinity or potency not determined.

Ligands Representatives	Sequence/Structure	Selectivity	Reference
***Agonists***			
***N*-Formyl peptides**			
**a.** **Bacteria-derived formyl peptides**			
fMLF	formyl-Met-Leu-Phe	FPR1 > FPR2	[40]
PSMα peptide	formyl-MGIIAGIIKFI KGLIEKFTGK	FPR2 > FPR1	[52]
**b.** **Mitochondria-derived formyl peptides**			
fMMYALF	formyl-Met-Met-Tyr-Ala-Leu-Phe	FPR1, FPR2	[53]
Mitocryptide-2	formyl-MTNIRKSHPLMKIIN	FPR2	[54]
**Non-formyl peptides**			
**a.** **Microbe-derived peptides**			
Hp2-20	AKKVFKRLEKLFSKIQNDK	FPR2 >> FPR3	[55]
**b.** **Host-derived peptides**			
SAA1.1	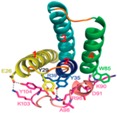	FPR2, others	[56,57]
Aβ42	DAEFRHDSGYEVHHQKLVFFAEDVGSNKGAIIGLMVGGVVIA	FPR2	[28,58]
Ac2–26	Ac-AMVSEFLKQAWFIENEEQEYVQTVK	FPR1, FPR2	[59,60]
LL-37	LLGDFFRKSKEKIGKEFKRIVQRIKDFLRNLVPRTES	FPR2	[61]
uPAR88-92	^88^Ser-Arg-Ser-Arg-Tyr^92^ (SRSRY)	FPR1	[62]
uPAR84-95	AVTYSRSRYLEC	FPR2, FPR3	[63]
PrP(106-126)	KTNMKHMAGAAAAGAVVGGLG	FPR2	[64]
SHAAGtide	MLWRRKIGPQMTLSHAAG	FPR2 > CCR1	[65]
VIP	HSDAVFTDNYTRLRKQMAVKKYLNSILN	FPR2, VPAC1	[66]
**c.** **Synthetic peptides**			
W peptides	WKYMVm(Trp-Lys-Tyr-Met-Val-D-Met-NH_2_)	FPR2 > FPR1 >> FPR3	[67,68,69]
	WKYMVM(Trp-Lys-Tyr-Met-Val-Met-NH_2_)	FPR2 >> FPR3	[69]
MMK1	LESIFRSLLFRVM	FPR2	[70]
L-37pA	DWLKAFYDKVAEKLKEAFPDWLKAFYDKVAEKLKEAF	FPR2	[71]
CGEN-855A	TIPMFVPESTSKLQKFTSWFM	FPR2, FPR3	[72]
**d.** **Pepducins**			
F2Pal_16_	Pam-KIHKKGMIKSSRPLRV	FPR2	[72,73]
**Eicosanoids**			
Lipoxin A4	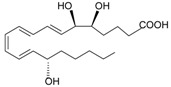		[74]
**Small molecules**			
**a.** **Quinazolinones** Quin-C1	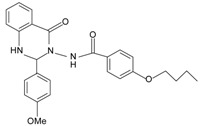	FPR2 >> FPR1	[75]
**b.** **Benzimidazoles** AG-09/1	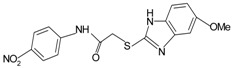	FPR1	[76]
**c.** ***N*-substituted benzimidazole derivative** *N*-substituted benzimidazole 11	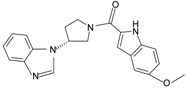	FPR2	[77]
**d.** **Pyrazolones** Compound **43**	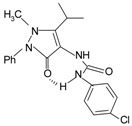	FPR2 > FPR1	[78,79]
**e.** **Pyridazin-3(2*H*)-ones** Pyridazin-3(2*H*)-one derivative 1	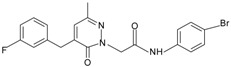	FPR1	[80]
Pyridazin-3(2*H*)-one derivative 2	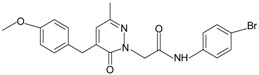	FPR2	[80]
**f.** ***N*-phenylureas** AG-26	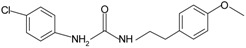	FPR2	[76]
**g.** **Chiral pyridazines**	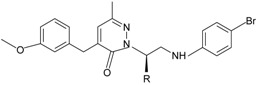 R = *n*-C_3_H_7_, *i*-C_3_H_7_, *n*-C_4_H_9_	FPR1, FPR2	[81]
**h.** **2-(N-Piperazinyl) acetamide derivatives** AG-09/73	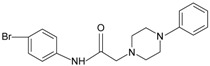	FPR2	[76]
**i.** **chiral 3-(1H-indol-3-yl)-2-[3-(4-nitrophenyl)ureido]propanamides** PD176252	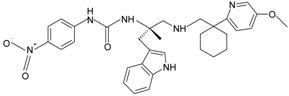	FPR2	[82,83]
**j.** **Others** 1753-103	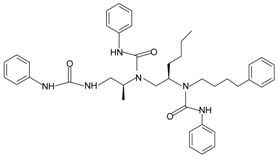	FPR1	[84]
***Antagonists***			
**Peptides**			
**a.** **Natural peptides & analogs**			
Boc-1	*N*-*tert*-butoxycarbonyl-MLF	FPR1 >> FPR2	[40]
Boc-2	*N*-tert-butoxycarbonyl-FLFLF	FPR1 >> FPR2	[40]
CHIP peptide Uteroglobin (UG)	FTFEPF MKLAVTLTLVTLALCCSSASAEICPSFQRVIETLLMDTPSSYEAAMELFSPDQDMREAGAQLKKLVDTLPQKPRESIIKLMEKIAQSSLCN	FPR1 FPR2	[85] [86]
Cyclosporin H (CsH)	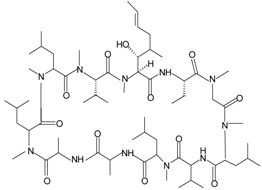	FPR1	[87]
Cyclized uPAR88-92	[^88^Ser-Arg-Ser-Arg-Tyr^92^]( cyclized SRSRY)	FPR1, N.D for FPR2	[88]
**b.** **Synthetic peptides**			
WRW^4^	WRWWWW (Trp-Arg-Trp-Trp-Trp-Trp)	FPR2, FPR3	[89]
PBP10	RhoB-QRLFQVKGRR	FPR2, others	[90]
**Lipopeptides**			
F1Pal_16_	Pam-KIHKQGMIKSSRPLRV	FPR2	[91]
Pam-(Lys-βNSpe)_6_-NH_2_	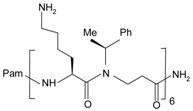	FPR2, mFpr2	[92,93]
**Non-peptide molecules**			
Isoflavone analog	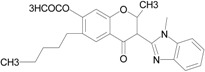	FPR1	[94]
1754-31	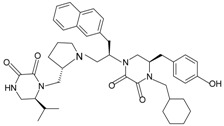	FPR2	[84]
Quin-C7	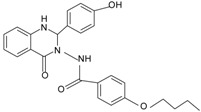	FPR2	[95]

## Abbreviations

The following abbreviations are used in this manuscript:

AD	Alzheimer’s disease
AhR	aryl hydrocarbon receptor
Aβ	β-amyloid
BLT1	Leukotriene B4 receptor 1
CB1	cannabinoid receptor type 1
C5aR	complement component 5a receptor
CDCA	chenodeoxycholic acid
CHIPS	Chemotaxis inhibitory protein of *S. aureus*
CHO	Chinese hamster ovary
CsA	Cyclosporin A
CsH	Cyclosporin H
CXCR1	C-X-C motif chemokine receptor 1
CXCR4	C-X-C chemokine receptor type 4
DAMP	damage-associated molecular pattern
DCA	deoxycholic acid
EC_50_	half maximal effective concentration
*E. coli*	*Escherichia coli*
FAM3D	family with sequence similarity 3 member D
FPR	Formyl peptide receptor
GPCR	G protein-coupled receptor
*H. pylori*	*Helicobacter pylori*
HTS	high throughput screening
*i*-BOC	*iso*-butyloxycarbonyl
IC_50_	half maximal inhibitory concentration
*K*_d_	dissociation constant
*K*_i_	inhibitor constant
*L. monocytogenes*	*Listeria monocytogenes*
LXA4	lipoxin A4
μOR	μ-opioid receptor
oxLDL	oxidized low-density lipoprotein
PAMP	pathogen-associated molecular pattern
P2Y_2_R	P2Y purinoceptor 2
ROS	reactive oxygen species
SAA	serum amyloid A
SAR	structure-activity relationships
*S. aureus*	*Staphylococcus aureus*
*t*-Boc	*tert*-butyloxycarbonyl
uPA	Urokinase plasminogen activator
UG	Uteroglobin
